# Application of ultrasound multimodal score in the assessment of endometrial receptivity in patients with artificial abortion

**DOI:** 10.1186/s13244-020-0840-5

**Published:** 2020-03-02

**Authors:** Yan Jiao, Nianyu Xue, Xujuan Shui, Caicha Yu, Chunhong Hu

**Affiliations:** 1 Department of Radiology, The First Affiliated Hospital of Soochow University, No. 188 Shizi Street, Suzhou, 215006 China; 2 Obstetrics and Gynecology Ultrasonic Department, Wenzhou Peoples’ Hospital, Wenzhou, 325000 China; 3 Department of Diagnostic Ultrasonography, Ningbo First Hospital, Ningbo, 315010 China

**Keywords:** Endometrial receptivity, Artificial abortion, Ultrasonic multimode, Quantitative scores

## Abstract

**Background:**

This study aimed to evaluate the value and feasibility of ultrasound multimodal score in the evaluation of endometrial receptivity in patients with artificial abortion (AA).

**Methods:**

Sixty-eight patients with AA (AA group) and 70 women of the childbearing age without any history of abortion (control group) were recruited between January 2018 and December 2018. All subjects received the examination of endometrium in the middle luteum phase (7–9 days after ovulation) with two-dimensional gray-scale ultrasound, two-dimensional color Doppler ultrasound, and three-dimensional ultrasound, and the quantitative scores were obtained and compared between two groups.

**Results:**

The quantitative score of endometrial receptivity was 10.46 ± 2.99 in the AA group and 13.49 ± 2.21 in the control group showing significant difference (*p* < 0.05).

**Conclusions:**

Ultrasound multimodal quantitative scores can be used to evaluate the endometrial receptivity of patients with AA.

## Key points

- Multimodal ultrasound can effectively evaluate some endometrium-related factors such as endometrial morphology, blood flow, and peristaltic waves.
- The ultrasound multimodal scoring is based on some features of the endometrium and may provide a more comprehensive reflection of endometrial receptivity.
- In the future, this scoring system has the potential to assist the reproductive physician to more accurately predict the outcome of pregnancy and plan the treatment.

## Background

In recent years, the prevalence of artificial abortion is increasing and has become an important public health problem. Abortion can cause mechanical damages to the endometrium, which may lead to secondary infertility, seriously affecting the physical and mental health and quality of life [1]. Studies have shown that secondary infertility may be related to the alteration of endometrial receptivity [2], and thus, it is important to evaluate the endometrial receptivity after artificial abortion. A variety of studies have suggested that ultrasound can be used to assess the endometrial receptivity [3]. However, few studies were conducted to evaluate the endometrial receptivity in patients with induced abortion by using ultrasound multimodal score [4]. This study aimed to evaluate the clinical value of ultrasound multimodal score in the evaluation of endometrial receptivity in patients with induced abortion.

## Materials and methods

### General characteristics

Patients who had not given birth but received artificial abortion of one to three times were recruited as the artificial abortion (AA) group (*n*=68) between January 2018 and December 2018. In addition, 70 subjects of the childbearing age who had not given birth and no history of artificial abortion were recruited as the control group in the same period. The inclusion criteria were as follows: Patients were ≥ 23 years, but ≤ 35 years; the menstrual cycle was regular within 1 year (28–35 days); the thyroid function was normal; patients were negative for abortion-related immune antibodies; the periodicity of sexual hormones was normal; the follicular hormone level was normal in the early follicular phase; the chromosomes of the father, the mother, and the infant were normal; ultrasonography of the uterus, uterine appendages, and pelvis showed normal; organic diseases and infectious diseases were excluded; there were no gynecological surgeries within prior 2 months; there were no medications affecting the hormones; and the semen analysis showed normal. The signed informed consent was obtained from each subject. The height and weight were measured for the calculation of body mass index (BMI). On the day of ultrasound examination, blood was collected to detect the estradiol, progesterone, testosterone, luteinizing hormone, prolactin, and follicle-stimulating hormone.

### Ultrasonography

#### Determination of implantation window phase

The follicles were examined by ultrasonography since the 10th day of the menstrual cycle. The size of follicles was observed in the peri-ovulation phase until ovulation (follicle rupture or disappearance). If ovulation was not observed, the examination was abandoned. Implantation window phase was defined as 7–9 days after ovulation when ultrasonography was performed. In AA group, ultrasonography was performed at 3 months after the last artificial abortion.

#### Ultrasound examination

Color Doppler ultrasound system (GE Voluson E8) with vaginal probe (RIC6-12-D) at the frequency of 5–8 MHz was used for ultrasound examination. Patients lied in a lithotomy position. The vaginal probe was wrapped with a condom, smeared with coupling agents, and then placed into the vagina. Measurement was done at the vaginal fornix.

#### Two-dimensional gray-scale ultrasound mode

Measurement of endometrial thickness: The distance between the interface of anterior wall muscle and the intima and the interface of posterior wall muscle and the intima was measured at 2 cm from the bottom of the uterine in the middle long axis view of the uterus. Measurement was done thrice and the mean was calculated. < 7 mm or > 14 mm was given 1 point, 7–8 mm was given 2 points, and 9–14mm was given 3 points.

**Fig. 1:**
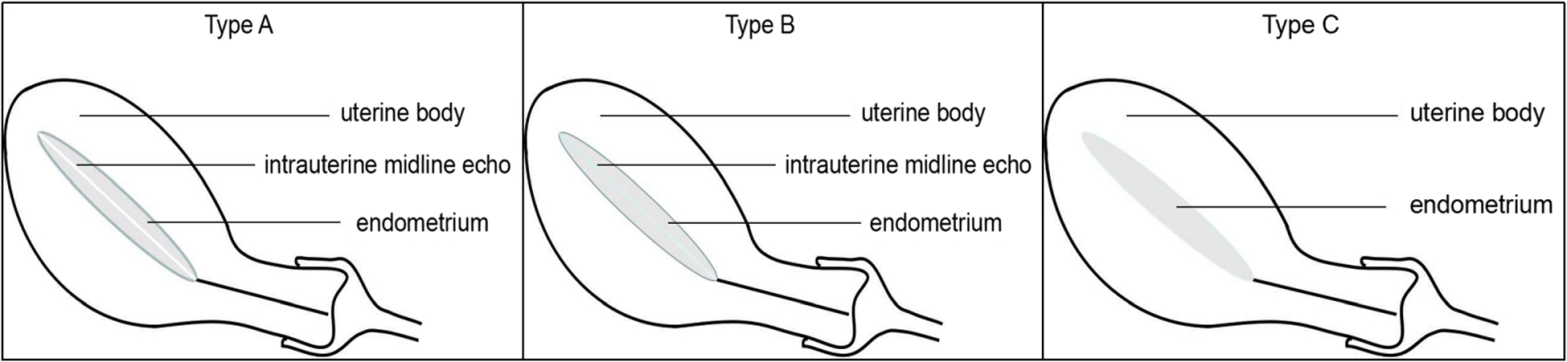
Endometrial patterns based on the Gonen system. Type A: trilaminar pattern (endometrial three-layer pattern) consisting of hyperechoic outer and middle layers, hypoechoic inner layers, and evident echo at the intrauterine midline; type B: relatively homogeneous hyperechoic endometrium, with unclear endometrial layers, obscure intrauterine midline echo, but clear interface between endometrial and muscular layers; type C: homogeneous hyperechoic endometrium without intrauterine midline echo

Observation of endometrial morphology: The echoes of the endometrium was observed. Then, the endometrial patterns were divided into three types based on the Gonen system: type A, trilaminar pattern (endometrial three-layer pattern), consisting of hyperechoic outer and middle layers, hypoechoic inner layers, and evident echo at the intrauterine midline; type B, relatively homogeneous hyperechoic endometrium, with unclear endometrial layers, obscure intrauterine midline echo, but clear interface between endometrial and muscular layers; and type C, homogeneous hyperechoic endometrium without intrauterine midline echo [5] (Fig. 1). Type C was given 1 point, type B was given 2 points, and type A was given 3 points.

Observation of endometrial movement: In the middle long axis view of the uterus, the position of the probe was fixed, and the patient was asked to calmly breathe. The type and frequency (times/min) of endometrial wave peristalsis were recorded for 3 min. Ljland et al. divided the endometrial peristalsis into 5 types: (1) positive wave, the peristaltic wave from the cervix to fundus; (2) negative wave, the peristaltic wave from the fundus to the cervix; (3) static wave, the endometrium in a static state; (4) bidirectional wave, the endometrium at the fundus and the cervix contract simultaneously; and (5) local peristaltic wave, the wave with low amplitude, no obvious directionality and rhythm [6]. Negative wave, static wave, bidirectional wave, and local peristaltic wave were given 1 point, positive wave < 1/min or > 3/min were given 2 points, and positive wave 1–3/min were given 3 points.

#### Two-dimensional color Doppler ultrasound examination

**Fig. 2:**
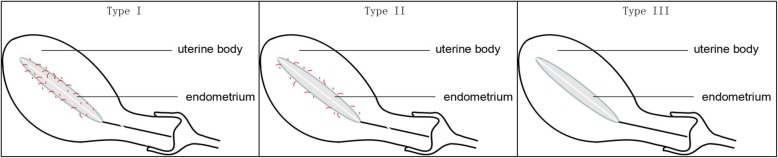
Endometrial blood flow patterns. Red lines indicate blood flow. Type I: blood flow is present in the endometrium and subendometrium; type II: blood flow is absent in the endometrium, but present in the subendometrium; type III, blood flow is not observed in both endometrium and subendometrium

Observation of blood flow distribution in the intima and subintima: In the meddle long axis view of the uterus, the subintimal area was defined as the area 3 mm away from the intimal edge, and the endometrial and subendometrial perfusion was detected with CDFI mode and classified as 3 types: type I, blood flow is present in the endometrium and subendometrium; type II, blood flow is absent in the endometrium, but present in the subendometrium; and type III, blood flow is not observed in both endometrium and subendometrium [7] (Fig. 2). Type III was given 1 point, type II was given 2 points, and type I was given 3 points.

#### Three-dimensional ultrasound examination

Measurement of endometrial volume and vascularization flow index: The energy Doppler blood flow imaging mode was used for measurement at a sensitive state. The branching of endometrial blood flow was assessed. Then, the 3D mode was set, VOCAL software was used for analysis, the endometrium was outlined, and then the endometrial volume (EV) and vascularization flow index (VFI) were determined [3, 8–10]. EV < 2 cm^3^ was given 1 point, EV of 2–4 cm^3^ was given 2 points, and EV > 4 cm^3^ was given 3 points. Unmeasurable VFI was given 1 point, VFI < 0.24 was given 2 points, and VFI > 0.24 was given 3 points.

#### Endometrial ultrasound score

As shown in Table 1, the endometrial thickness, endometrial type, endometrial peristalsis, endometrial and subendocardial blood flow distribution, EV, and VFI were scored in each subject, and the sum of these scores in each subject was used as the ultrasound multimodal score.

**Table 1:** Criteria for endometrial ultrasound scoring

Parameter	1 point	2 points	3 points
Endometrial thickness (mm)	< 7 or > 14	7–8	9–14
Endometrial type	C	B	A
Endometrial peristalsis (per min)	Negative wave or other types	Positive < 1 or > 3	Positive 1–3
Endocardial and subendocardial blood flow distribution	III	II	I
EV (cm^3^)	< 2	2–4	> 4
VFI	Immeasurable	< 0.24	> 0.24

### Statistical analysis

Statistical analysis was performed with SAS 9.4 (SAS Institute Inc., Cary, NC, USA). Normal distribution of data was tested with one-sample Kolmogorov-Smirnov test, and normal distribution was defined once *p* > 0.05 was observed. Data with normal distribution were compared with Tukey test between two groups. Data with abnormal distribution were compared with Kruskal-Wallis rank sum test, followed by Dwass-Steel-Critchlow-Fligner (DSCF) test between two groups. A value of *p* < 0.05 was considered statistically significant.

## Results

All the data acquired in two groups showed normal distribution.

General characteristics: There were no significant differences in the age, BMI, and other demographics between two groups (*p* > 0.05) (Table 2).

**Table 2:** Different parameters in two groups

Group	*n*	Age (years)	BMI (kg/m^2^)	Estradiol (ng/mL)	Progesterone (pg/mL)	Testosterone (nmol/L)	Luteinizing hormone (mIU/mL)	Prolactin (nmol/L)	Follicle-stimulating hormone (mIU/mL)	Endometrial ultrasound multimodal score
AA	68	24.79 ± 4.47	21.30 ± 4.21	300.88 ± 41.06	8.81 ± 2.19	2.31 ± 0.55	68.17 ± 13.41	0.58 ± 0.15	14.59 ± 3.86	10.46 ± 2.99
Control	70	25.38 ± 3.48	21.72 ± 3.47	300.17 ± 32.00	8.69 ± 1.88	2.26 ± 0.47	69.16 ± 10.41	0.57 ± 0.11	15.69 ± 3.78	13.49 ± 2.21^*^
*t* *P*		0.863 0.389	0.639 0.524	0.113 0.910	0.345 0.731	0.573 0.567	0.483 0.630	0.446 0.657	1.691 0.093	6.754 0.000

Note: *p < 0.05 vs AA group

Hormone levels in two groups: Significant differences in the levels of progesterone, estradiol, testosterone, luteinizing hormone, prolactin, and follicle-stimulating hormone were not observed between two groups (*p* > 0.05) (Table 2).

**Fig. 3:**
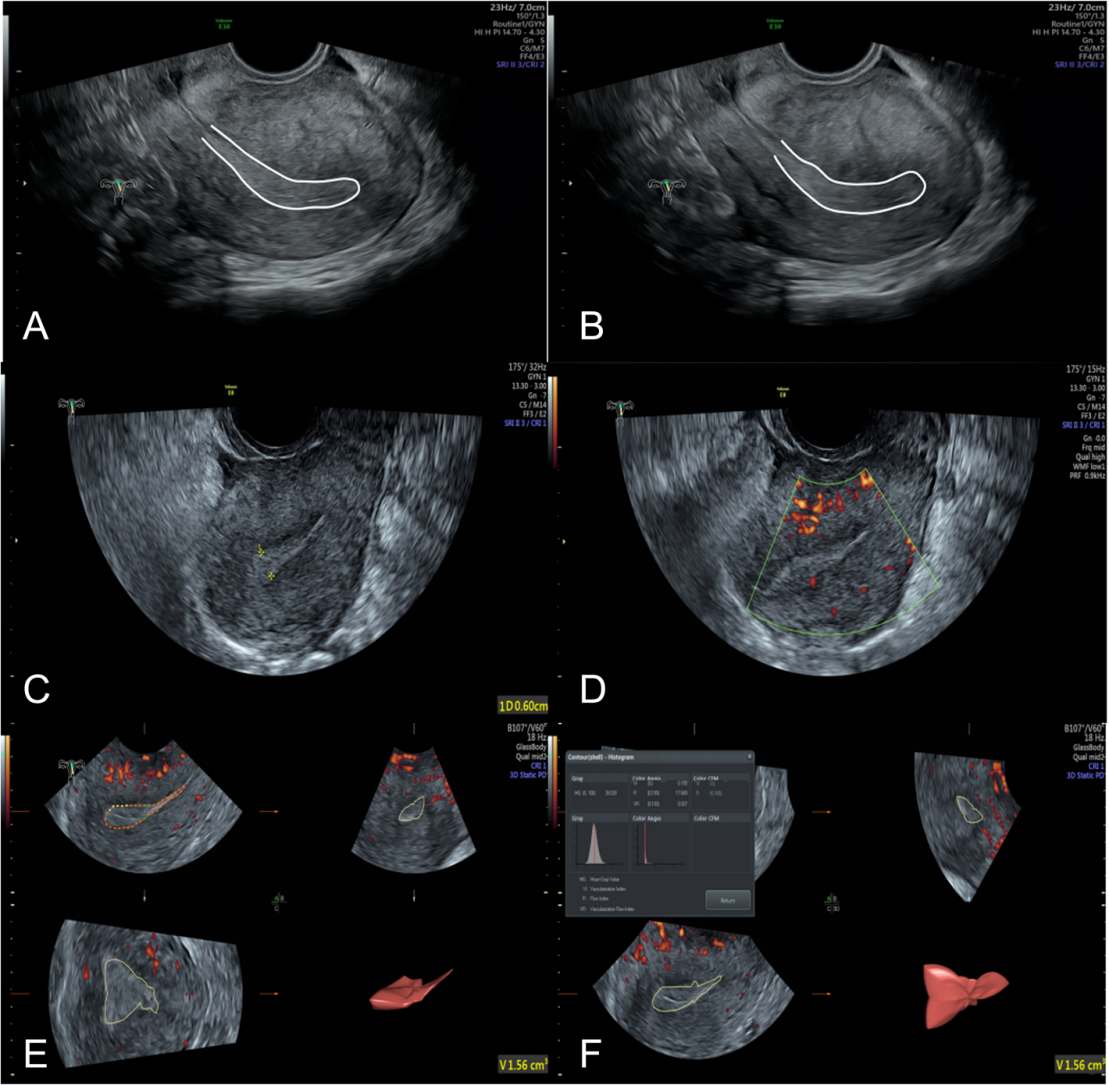
Ultrasound images of a subject in the AA group. **a**, **b** Two-dimensional gray-scale mode; two images during observation. White curve, outline of endometrium. No endometrium peristalsis wave within 3 min; score, 1; **c** two-dimensional gray-scale mode; endometrial thickness, 6.0 mm; score, 1; endometrial type, type C; score, 1; **d** 2D color Doppler mode; a little blood flow in the subendometrium; score, 2; **e** three-dimensional mode; EV, 1.56 cm^3^; score, 1; **f** three-dimensional mode; VFI, 0.027; score, 2

**Fig. 4:**
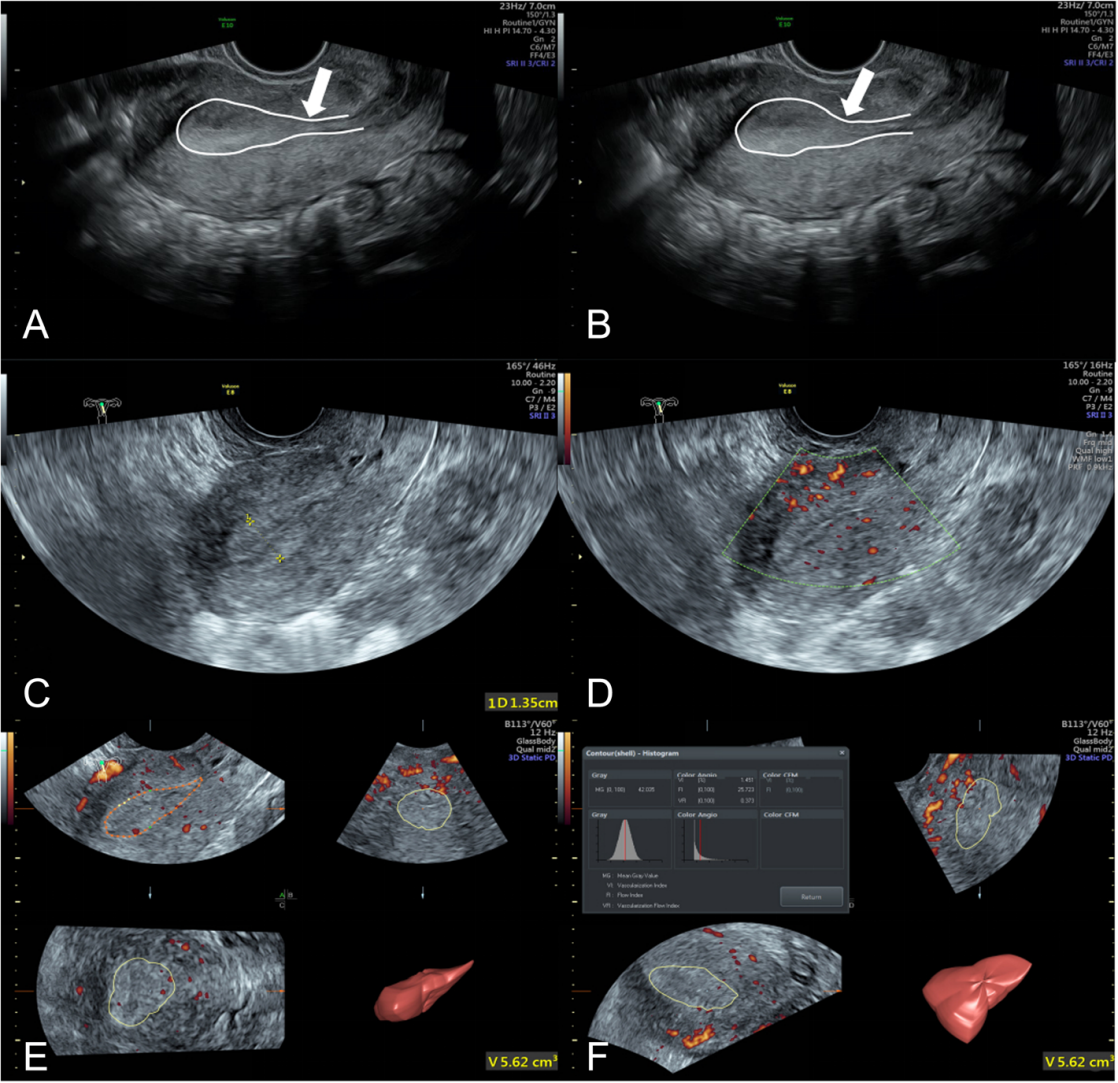
Ultrasound images of a subject in the control group. **a**, **b** Two-dimensional gray-scale mode; two images of one peristalsis wave during observation. White curve, outline of endometrium. White arrow, incisura of peristalsis wave. Six positive endometrium peristalsis wave within 3 min (2/min); score, 3; **c** two-dimensional gray-scale mode; endometrial thickness, 13.5 mm; score, 3; endometrial type, type A; score, 3; **d** two-dimensional color Doppler mode; endometrial and subendocardial blood flow were observable; score, 3; **e** three-dimensional mode; endometrial volume, 5.62 cm^3^; score, 3; **f** three-dimensional mode; VFI, 0.373; score, 3

Endometrial ultrasound multimodal score: The ultrasound multimodal score was 10.46 ± 2.99 in the AA group and 13.49 ± 2.21 in the control group. The ultrasound multimodal score in the AA group was markedly lower than in the control group (*p* < 0.05) (Table 2) (Figs. 3 and 4).

## Discussion

Artificial abortion may inevitably damage the endometrium and other organs in the reproductive system [11, 12]. In addition, artificial abortion also has the potential to cause some complications such as secondary abortion, abnormal placenta, premature delivery, and ectopic pregnancy [13]. Therefore, endometrial repair is the most important for the post-abortion rehabilitation. At present, the clinical manifestations such as time of postoperative bleeding, blood loss, and time to recovery of menstrual cycle as well as the endometrial thickness are monitored for the assessment of endometrial repair, but these features have limitations in the monitoring of disease condition and the diagnosis and treatment of diseases. In recent years, increasing attention has been paid to the role of recovery of endometrial receptivity in the endometrial repair [4, 14–16]. Although many studies have been conducted to investigate the endometrial receptivity, the methods in these studies are often different and there are no widely accepted criteria for the assessment of endometrial receptivity. In this study, a new ultrasound multimodal endometrial scoring system was developed based on the findings in previous studies. The total score was 18 in our scoring system. The higher the score, the better the endometrial receptivity, and vice versa [17, 18]. Ultrasound multimodal score used in this study included endometrial thickness, endometrial type, endometrial peristaltic wave, blood flow, volume, and VFI, which can be easily acquired with present ultrasound technology and universally accepted in clinical practices. Besides, every indicator was acquired with a universal measuring method, and the scoring of each indicator adopted a universal standard. What is more, there were still requirements for the operators that they have to be skilled to acquire the above indicators. Meanwhile, the measuring method and scoring standard must be conducted according to the rules set in this study. This was not hard for experienced sonographer of gynecology. Above measures will reduce the influence of subjective judgement of sonographer on ultrasound multimodal score furthest.

In the present study, results showed the endometrial ultrasound multimodal score was 10.46 ± 2.99 in the AA group and 13.49 ± 2.21 in the control group. The endometrial ultrasound multimodal score in the AA group was significantly lower than in the control group. This indicates that, compared with normal controls, the endometrium of patients with artificial abortion have one or more abnormalities (morphology, blood flow, and peristaltic wave), which reduces the total ultrasound multimodal score. This may be related to the pathological responses of the endometrium (significant reduction in the endometrial glandular epithelium and marked increase in collagen fibers) after the mechanical injury to the endometrium [19–22]. These pathological responses may dramatically increase the proportion of collagen fibers in the endometrium and, as a result, the blood flow in the endometrium reduces, the endometrium becomes thinner, and the EV reduces. Studies have shown that the endometrial thickness, EV, VFI, and other parameters in abortion patients are significantly lower than in normal pregnant subjects [23–25]. Most previous studies about endometrial receptivity evaluated one or some of the indicators including endometrial thickness, endometrial type, endometrial peristaltic wave, blood flow, volume, and VFI. Actually, each indicator could reflect different characteristics of endometrium, respectively. That is to say more indicators could evaluate the endometrium more comprehensively. However, there is still a need of considering feasibility in clinics. This study adopted six typical indicators, not only fully reflecting the endometrium situation, but also not bringing difficulties to clinical practices. These indicators still had a relatively big feasibility and popularization possibility for not only ultrasonic technique but also operators at present. In the present study, two-dimensional gray-scale ultrasound, two-dimensional color Doppler ultrasound, and three-dimensional ultrasound were employed simultaneously to assess the endometrial morphological, structure, and blood flow, which is helpful for the comprehensive evaluation of endometrial receptivity. This can effectively compensate for the disadvantage of individual factor in the assessment of endometrial receptivity. In addition, studies have indicated that endometrial receptivity is affected by the sexual hormones and age [26, 27]. In the present study, there were no significant differences in the age and BMI between AA group and control group. In addition, marked differences were also not observed in other hormones such as progesterone, estradiol, testosterone, luteinizing hormone, prolactin, and follicle-stimulating hormone. This indicates that the difference in the endometrial receptivity between the AA group and the control group is mainly ascribed to the endometrial state, but not the age and hormones. The ultrasound modes used in this study have been well popularized in clinical practice. Therefore, the method used in the present study is a simple, safe, non-invasive, and reproducible tool for the assessment of endometrial receptivity and deserves clinical properties [2].

There were still limitations in this study. There are no widely accepted criteria or consensuses on the multimodal ultrasound assessment of endometrial receptivity. In this study, a new system was developed for the assessment of endometrial receptivity on the basis of previous findings and it might be subjective in the development of this system. Therefore, it is necessary to further investigate the clinical value and practicality and to improve this scoring system.

## Conclusions

In summary, multimodal ultrasound can effectively evaluate some endometrium related factors such as endometrial morphology, blood flow, and peristaltic waves. The ultrasound multimodal scoring is based on some features of the endometrium and may provide a more comprehensive reflection of endometrial receptivity. In the future, this scoring system has the potential to assist the reproductive physician to more accurately predict the outcome of pregnancy and plan the treatment. However, the relationship and interaction of various factors are still unclear, and more studies are needed to confirm our findings.

## Data Availability

The datasets used and/or analyzed during the current study are available from the corresponding author on reasonable request.

## Abbreviations

AA: Artificial abortion
BMI: Body mass index
DSCF: Dwass-Steel-Critchlow-Fligner
EV: Endometrial volume
VFI: Vascularization flow index
